# Mortality associated with Sjögren’s syndrome in the United States in the 1999–2020 period: A multiple cause-of-death study

**DOI:** 10.1515/med-2023-0829

**Published:** 2023-10-30

**Authors:** Rongxiu Huo, Xinxiang Huang, Jinying Lin

**Affiliations:** Department of Rheumatology and Immunology, Guangxi Academy of Medical Sciences, The People’s Hospital of Guangxi Zhuang Autonomous Region, Nanning City, 530016, China

**Keywords:** Sjögren’s syndrome, polymyalgia rheumatica, interstitial lung disease, tumor, heart diseases

## Abstract

The study aimed to analyze the mortality and leading causes of death associated with Sjögren’s syndrome (SS) in the United States (US) between 1999 and 2020 using a multicause approach. We analyzed mortality based on SS as the cause-of-death. Using mortality rates, number of deaths, and historical trends, we examined sex, age of death, comparisons of SS- and polymyalgia rheumatica-related deaths (multiple cause-of-death) in the last 20 years, changes in the ranking of causes of death when SS was the underlying cause-of-death (UCD) in the first and last 5 years of the last 20 years, and the number of deaths and standardized mortality (per 100,000 people) when SS combined with interstitial lung disease (ILD) or tumor was the multiple cause-of-death. An SS-standardized mortality trend chart and a trend line were created. In 22 years, the total number of SS-related deaths in the US was 7,817, including 7,016 women. When SS was the UCD and non-UCD, the standardized ratios of female-to-male deaths (per 100,000 people) were approximately 4.6–13:1 and 6.8–19.6:1, respectively. SS-related deaths were more common in people aged >60 years and concentrated in patients aged 60–79. In cases where SS and polymyalgia rheumatica were the multiple cause-of-death, the number of deaths and age-standardized mortality of SS and polymyalgia rheumatica increased, although lower in SS than in polymyalgia rheumatica. Regarding SS as the UCD, heart disease ranks first. Concerning the number of deaths and standardized mortality in the first (1999–2003) and second (2016–2020) 5 years, when SS-ILD and SS combined with tumors were the multiple causes of death, the number increased in the second 5 years compared to that in the first 5 years. When SS combined with COVID-19 was the multiple cause-of-death, 73 deaths occurred, comprising 64 females and 9 males. Death predominance was observed among women and patients aged 60–79 years with SS. Although the SS-standardized mortality rate was low, an increasing trend was observed. When SS was the primary cause-of-death, heart disease remained primarily involved, followed by malignant neoplasms. The number of patients with SS-ILD and SS combined with tumors in the past 22 years and the standardized mortality rate after 5 years increased compared with those of the previous 5 years. Concurrent SS and COVID-19 may be related to the increased SS deaths.

## 1 Introduction

Sjögren’s syndrome (SS) is a chronic autoimmune disease characterized by dryness of the eyes and mouth caused by lacrimal and salivary gland dysfunction. It primarily affects women aged 4–60 years, with an average male-to-female ratio of approximately 1:10 [1]. The estimated incidence and prevalence of SS were 6.92/100,000 and 60.82/100,000 persons/year, respectively [1]. The estimated 5- and 10-year survival rates after SS diagnosis are 95 and 90%, respectively [2], and the overall mortality within 7 years is twice that of the general population [3]. Research shows that the standardized mortality rate is 0.85–1.38 [4,5]. Similarly, polymyalgia rheumatica is a rheumatic disorder associated with moderate-to-severe musculoskeletal pain and neck, shoulder, and hip stiffness. Its incidence increases with age [6]. In different populations, the prevalence rate is 12–60/100,000 people, and the standardized mortality rate is approximately 0.70 [7,8], similar to that of SS; however, relevant research on comparing the two is lacking.

SS can involve extraglandular organs (including the skin, lungs, and blood system) and can be complicated by interstitial lung disease (ILD) and tumors. SS-ILD is considered mild and noninvasive; the average survival time after its diagnosis is 9.0 (range: 6.8–13.0) years [9]. However, during 2- to 8-year follow-up, reported mortality rates range from 7.1 to 39% [9]. In addition, SS can be associated with tumor occurrence, which is a risk factor for SS mortality. Combined tumors include hematological (including lymphoma, leukemia, and multiple myeloma) and solid (including thyroid and breast cancers) tumors [10]. A study published in 2010 investigated tumors in SS and found that lymphoma was the most common, with an incidence rate of 5% [11].

Furthermore, a meta-analysis showed that cardiovascular diseases, tumors, and infections were the primary cause-of-death in patients with SS [4]. Studies have shown that death risk owing to cardiovascular or hematological malignancies is comparable between patients with SS and the general population [12]. However, whether the ranking of the cause-of-death mentioned in the SS has changed over the past 22 years is unclear.

Coronavirus disease 2019 (COVID-19) was first reported in Wuhan, Hubei Province, China, in December 2019. A study in the United Kingdom found that patients with rheumatism had a higher death risk owing to COVID-19 [13]. SS has specific characteristics associated with an increased risk of developing severe COVID-19 (autoimmune damage to the lungs, use of immunosuppressants, and lymphoma) [14,15,16]. Therefore, SS combined with COVID-19 can increase the proportion of patients with a poor prognosis, which is relatable to the increased SS-related deaths.

Therefore, this study aimed to describe SS-related deaths and their leading causes using a multicause approach based on data from death certificates obtained from the Centers for Disease Control and Prevention (CDC) over the last 22 years.

## 2 Methods

Using the United States (US) CDC database, we obtained the number of deaths per 100,000 people and the standardized mortality rate between 1999 and 2020. According to the 10th Revision of the International Classification of Diseases 10 (ICD-10) [17], the following are the category codes: SS (M35.0), polymyalgia rheumatica (M35.3), neoplasms (C00-D48), ILD (J84), and COVID-19 (U07.1). Using mortality rates, number of deaths, and historical trends, we considered the following variables: sex, age of death (<40, 40–59, 60–79 years), mortality of SS and polymyalgia rheumatica as multiple cause-of-death in the last 22 years, changes in the cause-of-death ranking when SS was mentioned as the underlying cause-of-death (UCD) in the first and last 5 years when SS was mentioned as the UCD, and the number of deaths and standardized mortality when SS was combined with ILD or tumors as multiple cause-of-death. The ratios of female-to-male deaths when SS was listed as the underlying and non-underlying causes were calculated separately. SS and polymyalgia rheumatica deaths and standardized mortality (per 100,000 people) were plotted using Microsoft Excel 2010 (Microsoft Corp., Redmond, WA, USA), and trend lines were added. Ethical approval: the conducted research is not related to either human or animal use.

## 3 Results

The total number of SS-related deaths in the US between 1999 and 2020 was 7,817, including 801 males (10.2%) and 7,016 females (89.8%) (Table 1), with a male-to-female ratio of approximately 1:9. When SS was listed as the UCD, the number of deaths was 2,149 (27.5%) during the period 1999–2020 and showed an increasing trend, with the number of male and female deaths increasing independently a decreasing standardized ratio of female to male deaths (per 100,000 people) of approximately 4.6–13:1, and SS deaths occurring more frequently in persons aged >60 years. It was concentrated in patients aged 60–79 years (Table 1). When SS was classified as a non-fundamental cause-of-death, 5,668 deaths (72.5%) occurred during the period 1999–2020, with an annual increase in the number of male and female deaths and a standardized ratio of approximately 6.8–19.6 deaths per 100,000 people for females and males. SS-related deaths were more common in patients aged 60–79 years (Table 1).

**Table 1:** Number of SS-related deaths in the US from 1999 to 2020 stratified by year

	1999	2000	2001	2002	2003	2004	2005	2006	2007	2008	2009	2010
All SS-related deaths, no.	267	273	269	276	272	254	274	267	270	299	258	331
**SS listed as the UCD**	70	75	70	80	84	66	90	85	78	100	72	94
Men, no.	9	7	5	7	8	7	9	11	9	10	7	9
Women, no.	61	68	65	73	76	59	81	74	69	90	65	85
**Ratio of women to men**	6.8	9.7	13	10.4	9.5	8.4	9	6.7	7.6	9	9.3	9.4
Age, no.												
<40 years	1	0	1	0	0	0	4	1	2	3	1	1
40–59 years	6	7	7	6	10	11	8	7	6	11	6	12
60–79 years	42	42	41	32	40	27	34	37	35	41	34	38
≥80 years	21	26	21	42	34	28	44	40	35	45	31	43
**SS listed as the NUCD**	197	198	199	196	188	188	184	182	192	199	186	237
Men, no.	24	15	14	16	17	15	16	14	21	14	24	24
Women, no.	173	183	185	180	171	173	168	168	171	185	162	213
**Ratio of women to men**	7.2	12.2	13.2	11.3	10.1	11.5	10.5	12	8.1	13.2	6.8	8.9
Age, no												
<40 years	2	3	4	3	3	1	4	2	2	3	1	2
40–59 years	17	19	20	25	23	28	20	22	23	23	19	23
60–79 years	105	92	99	105	95	87	83	83	76	94	81	108
>80 years	82	84	76	63	68	72	77	74	91	79	85	104

	2011	2012	2013	2014	2015	2016	2017	2018	2019	2020	All
All SS-related deaths, no.	350	348	405	405	400	427	412	489	582	689	7,817
**SS listed as the UCD**	103	96	112	120	111	117	107	136	146	137	2,149
Men, no.	10	10	11	12	16	13	15	22	26	21	254
Women, no.	93	86	101	108	95	104	92	114	120	116	1,895
**Ratio of women to men**	9.3	8.6	9.1	9	5.9	8	6.1	5.1	4.6	5.5	7.4
Age, no.											
<40 years	0	3	2	3	2	1	1	2	0	1	29
40–59 years	9	5	14	7	10	19	18	14	11	20	224
60–79 years	49	49	50	54	48	47	55	77	85	68	1,025
>80 years	45	39	46	56	51	50	33	43	50	48	871
**SS listed as the NUCD**	247	252	293	285	289	310	305	353	436	552	5,668
Men, no.	12	14	28	27	32	32	29	39	52	48	547
Women, no.	235	238	265	258	257	278	276	314	384	504	5,121
**Ratio of women to men**	19.6	17	9.5	9.6	8.0	8.7	9.5	8.1	7.4	10.5	9.4
Age, no.											
<40 years	2	1	5	3	6	3	2	0	3	9	62
40–59 years	27	34	28	31	39	40	34	43	44	48	630
60–79 years	113	118	145	126	131	157	160	157	198	286	2,699
>80 years	105	100	115	125	113	110	109	153	191	209	2,277

Abbreviations: UCD: underlying cause of death; NUCD: non-underlying cause of death; SS: Sjögren’s syndrome.

**Figure 1:**
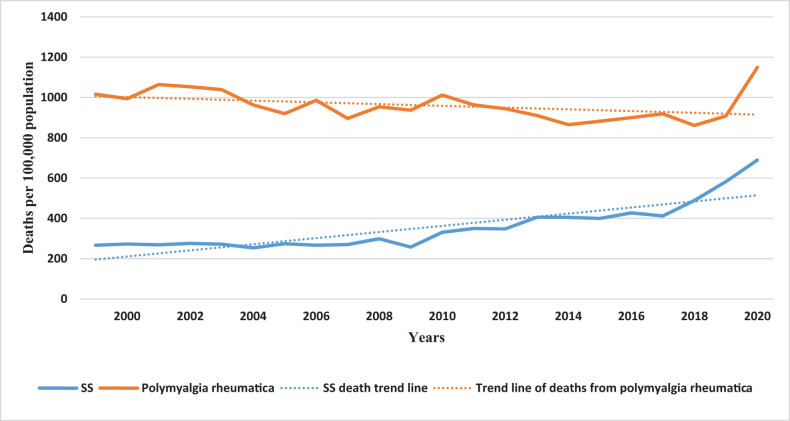
Trends in SS and polymyalgia rheumatica death toll per 100,000 in the US from 1999 to 2020. Abbreviations: SS: Sjögren’s syndrome.

**Figure 2:**
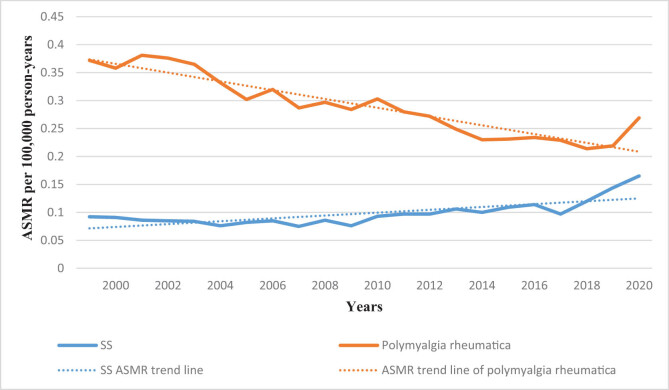
Trends in SS and polymyalgia rheumatica age-adjusted mortality rates per 100,000 in the US from 1999 to 2020. Abbreviations: SS: Sjögren’s syndrome; ASMR: age-standardized mortality rate.

We compared the period 1999–2020 SS and polymyalgia rheumatica as the deaths of multiple cause-of-death and found that the death toll in SS and polymyalgia rheumatica and age-standardized mortality rates (ASMRs) (per thousand population) were increasing, and the death toll from SS and standardized mortality rates (per thousand population) are lower than those of polymyalgia rheumatica (Figures 1 and 2).

Similarly, we investigated the ranking of the top 10 cause-of-death in the first (1999–2003) and last (2016–2020) 5 years over the past 22 years by mentioning SS as the root cause-of-death (Tables 2 and 3) and found that compared with the first 5 years, heart diseases and malignant neoplasms remained ranked at first and second, respectively, and diabetes mellitus was ranked seventh. Some diseases were newly discovered or changed in rankings, with COVID-19 and accidents (unintentional injuries) ranking fifth and ninth, respectively. Chronic lower respiratory diseases were ranked from fourth to third, and cerebrovascular diseases dropped from third to fourth place. Alzheimer’s disease ranked sixth from 10th, influenza and pneumonia fell to seventh place from 5th, and chronic liver disease and narrowing dropped from 6th to 10th place.

**Table 2:** Ranking of UCD in SS mentioned from 1999 to 2003

UCD – ICD-10 113 cause list	Deaths
#SS	1,357
#Diseases of heart (I00–I09, I11, I13, I20–I51)	216
#Malignant neoplasms (C00–C97)	157
#Cerebrovascular diseases (I60–I69)	68
#Chronic lower respiratory diseases (J40–J47)	51
#Influenza and pneumonia (J09–J18)	37
#Septicemia (A40–A41)	27
#Chronic liver disease and cirrhosis (K70, K73–K74)	22
#Diabetes mellitus (E10–E14)	17
#Nephritis, nephrotic syndrome and nephrosis (N00–N07, N17–N19, N25–N27)	14
#Alzheimer disease (G30)	13

Abbreviations: UCD: underlying cause of death; ICD-10: International Classification of Diseases 10.

**Table 3:** Ranking of UCD in SS mentioned from 2016 to 2020

UCD – ICD-10 113 cause list	Deaths
#SS	2,599
#Diseases of heart (I00–I09, I11, I13, I20–I51)	348
#Malignant neoplasms (C00–C97)	303
#Chronic lower respiratory diseases (J40–J47)	98
#Cerebrovascular diseases (I60–I69)	81
#COVID-19 (U07.1)	66
#Alzheimer disease (G30)	53
#Influenza and pneumonia (J09–J18)	47
#Diabetes mellitus (E10–E14)	43
#Accidents (unintentional injuries) (V01–X59, Y85–Y86)	37
#Chronic liver disease and cirrhosis (K70, K73–K74)	35

Abbreviations: UCD: underlying cause of death; ICD-10: International Classification of Diseases 10.

In addition, we compared the number of deaths and standardized mortality rates in the first (1999–2003) and second (2016–2020) 5 years when SS was combined as a multiple cause-of-death. We found that the total number of deaths in the first 5 years when SS-ILD was used as a multiple cause-of-death was 182. The standardized mortality rate was 0.013, the total number of deaths in the last 5 years was 419, the standardized mortality rate was 0.026, the number of deaths in the last 5 years was higher than that in the previous 5 years, and the standardized mortality rate increased (Tables 4 and 5).

**Table 4:** ILD deaths occurred in SS from 1999 to 2003

Year	Deaths	Crude rate
1999	33	0.012
2000	38	0.014
2001	36	0.013
2002	33	0.011
2003	42	0.014
1999–2003	182	0.013

**Table 5:** ILD deaths occurred in SS from 2016 to 2020

Year	Deaths	Crude rate
2016	81	0.025
2017	57	0.017
2018	70	0.021
2019	111	0.034
2020	100	0.03
2016–2020	419	0.026

When SS was combined with tumors as a multiple cause-of-death, the total number of deaths and the standardized mortality rates in the first and second 5 years were 241 and 0.017 and 451 and 0.028, respectively, with an increased number of deaths and standardized mortality rate in the second 5 years compared with those of the first 5 years (Tables 6 and 7). Furthermore, we found that when SS was combined with COVID-19 as a multiple cause-of-death, 73 deaths occurred in 2020, including 64 women (87.7%) and 9 men (12.3%). This represents 9% of the total SS-related deaths in the last 22 years, including 8% in women and 1% in men.

**Table 6:** Tumor deaths occurred in SS from 1999 to 2003

Year	Deaths	Crude rate
1999	56	0.02
2000	43	0.015
2001	52	0.018
2002	49	0.017
2003	41	0.014
1999–2003	241	0.017

**Table 7:** Tumor deaths occurred in SS from 2016 to 2020

Year	Deaths	Crude Rate
2016	80	0.025
2017	77	0.024
2018	85	0.026
2019	107	0.033
2020	102	0.031
2016–2020	451	0.028

## 4 Discussion

Our study showed that between 1999 and 2020, the standardized mortality rate of women with SS was consistently higher than that of men, with a mortality ratio of approximately 9:1. Higher SS-related mortality in women was associated with a higher prevalence in women. When SS was listed as the UCD and non-underlying cause-of-death (NUCD), the number of deaths increased, with a slight fluctuation in the number of deaths in males and an increasing trend in females, which may be related to the higher incidence in females [1] and the increase in the population base. In addition, studies have found that tolerable pathological syndromes are more common in women at the onset of SS [18]. Therefore, the delay in seeking medical treatment may involve multiple organs in the body, exacerbating the disease, possibly related to the increase in female mortality. SS becomes complex in patients from diagnosis with extraglandular involvement or related diseases and is characterized by significantly reduced survival rates, such as Raynaud’s phenomenon in women [18]. In men, extraglandular involvement may be the first symptom; therefore, early diagnosis can be determined, and timely treatment can be obtained [18] to control disease progression, which may result in a low mortality rate in men. Furthermore, we found that when SS was listed as the UCD or NUCD, the number of deaths increased in patients aged 40–59, 60–79, and >80 years; however, it primarily occurred in patients aged >60 years, particularly in patients aged 60–79 years, which may be related to the age at diagnosis. The average age at the first diagnosis of SS is reportedly 56 years [19]. A prospective study in Greece reported a higher death risk in patients with SS than in the general population [20]; however, the risks were similar in another study [21].

SS is a chronic, slow-developing, non-life-threatening disease in many patients, with a 10-year cumulative survival rate of >90%. The mortality rate of patients with SS is insignificantly higher than that of the general population [4]. Despite the seemingly favorable prognosis of SS, the number of deaths per 100,000 people and standardized mortality rate (fluctuating between 0.075 and 0.165) of SS in our study increased over the last 22 years (Figures 1 and 2). A previous meta-analysis reported a quasi-mortality rate of 1.38 in patients with SS [4]. Polymyalgia rheumatica has a good prognosis, and its prevalence rate is similar to that of SS. The number of deaths and age-standardized mortality of polymyalgia rheumatica in the past 22 years was higher than that of SS; however, the overall trend was downward, associated with better disease control in patients with polymyalgia. Although the standardized mortality rates of these diseases are lower than that of rheumatoid arthritis (1.65) [22], early diagnosis and treatment, disease activity control, and mortality reduction remain essential.

Tables 2 and 3 comprise the rankings of SS mentioned as the UCD in 1999–2003 and 2016–2020. Age-related diseases including heart diseases, malignant neoplasms, cerebrovascular diseases, and chronic lower respiratory diseases top the list. Previous studies believed that cardiovascular diseases and tumors were the primary cause-of-death [4]. The current addition, COVID-19, is related to the recent epidemic and the use of immunosuppressants in patients with SS, although hydroxychloroquine reportedly affects COVID-19 [23]. Accidents (unintentional injuries) are related to improving living standards and the widespread use of cars as transportation means. Influenza, pneumonia, chronic liver disease, and cirrhosis dropped from the top 5, and septicemia dropped out of the top 10 in the second 5 years. It is related to the recent upgrade of antibiotics, antiviral drugs, liver protection drugs, and the application of artificial livers [24,25,26].

As the disease progresses, patients with SS may develop comorbidities. The comorbidities may be related to the SS process and/or its treatment or completely independent. These comorbidities are associated with increased mortality rates in women and men. We primarily studied the more common comorbidities of SS, that is, the cases of SS-ILD and SS combined with tumors in the last and the first 5 years in the past 22 years and the number of deaths caused by SS combined with COVID-19 in 2020. Different comorbidities act differently in females with SS, and progressive dyspnea and respiratory failure may occur when combined with lung lesions (such as ILD and pulmonary hypertension) [27]. When combined with infection [28], sepsis and septic shock may occur in severe cases. When combined with malignant tumors [5], cachexia may occur, and when it metastasizes to vital organs (intracranially), cerebral herniation may occur. These complications might cause higher mortality rate in females with SS.

Pulmonary involvement is a common extraglandular complication, with a recently reported prevalence of 16%, and is reportedly associated with poor prognosis and higher mortality in patients with SS [29]. Studies have reported that ILD is associated with decreased functional status in patients with SS, and 36.8% of patients with SS-ILD have significantly reduced activity owing to worsening ILD, causing respiratory failure in 11% of cases [30]. Palm et al. [31] reported a four-fold increased risk of death after 10 years in patients with SS-ILD. Although the 10-year survival rate for patients with SS-ILD was 81.7% [9], our results showed that deaths from SS-ILD as a multiple cause-of-death increased, and the standardized mortality rate increased in 2016–2020 compared with that of 1999–2003 (Tables 4 and 5). This finding may be related to patient age. Age is a risk factor for death, and ILD deterioration is more common in older aged patients with SS at ILD diagnosis and slightly related to inflammatory status and autoantibodies [30]. Therefore, clinicians should assess and monitor lung involvement, particularly in patients without pulmonary manifestations.

SS is strongly associated with an increased risk of developing tumors, particularly lymphoma, which is 10–44 times higher than that in healthy individuals and higher than that reported for systemic lupus erythematosus (seven times) and rheumatoid arthritis (four times) [32]. The standardized incidence (SIR) estimates for all tumors were 1.91 (95% CI, 1.60–2.28). The SIR for solid tumors and blood cancers was 1.13 (95% CI, 0.88–1.46) and 11.02 (95% CI, 8.35–14.54), respectively [10]. Our results showed that when SS combined with tumors was a multiple cause-of-death, deaths increased amongst patients, and the standardized mortality increased in the last 5 years compared with that of the first 5 years in the past 20 years; this may be related to age. Studies have found that the mean age at diagnosis of SS combined with tumor was 55.1 (SD 15.4) (range, 15.3–92.9) years [10]. Additionally, studies have confirmed that severe parotid involvement, purpura, leukopenia, anti-LA antibodies, elevated levels of β2 microglobulin, cryoglobulin, and hypocomplementemia are risk factors for cancer [10,33,34,35]. Therefore, physicians should observe the clinical manifestations of SS in patients aged >50 years and periodically review the relevant indicators. When changes occur, observing whether the tumor is combined is essential.

Furthermore, when SS combined with COVID-19 was a multiple cause-of-death, 73 deaths occurred in 2020 alone, 9% of the total SS-related deaths in the last 22 years. This may be related to the potential abnormal immune response and frequent use of immunosuppressants in patients with SS, causing a high hospitalization rate and poor prognosis [36], including respiratory failure and esophageal lymphocytosis, leading to patient death. Studies have shown increased risks of hospitalization and death, particularly in patients treated with corticosteroids [37].

We re-examined SS-related mortality over the past 22 years. Using multiple cause-of-death analysis is a primary feature and strength of this study. Multiple cause-of-death analyses have been used to determine the maximum possible number of deaths associated with autoimmune diseases, often referred to as related cause-of-death [38]. In this study, SS was classified as a related cause of 72.5% of deaths. The underestimation of rheumatic diseases in mortality statistics is attributed to identifying one of their complications, such as infection or cardiovascular disease, which often become the UCD.

This study has certain limitations. First, despite the automated processing of mortality data used in the US, including the cause of death, the study was conducted by trained disease logists who might have committed errors and introduced incorrect ICD-10 codes. Second, there were no search terms for adherence to therapy and marital status in the system, which could not be further analyzed. At the same time, the number of deaths was relatively small, and subgroup analysis of patients with various complications associated with SS could not be performed.

## 5 Conclusion

In this study, we used multiple cause-of-death analyses to characterize SS-related mortality in the US over nearly 22 years. We observed death predominance among women and older adults. Similarly, the standardized mortality rate for SS increases and that only 27.5% of deaths were identified as the underlying cause of SS. This suggests that the SS mortality statistics may be underestimated. In contrast, the age-standardized mortality in patients with polymyalgia rheumatica showed a downward trend. When SS was mentioned as the primary cause-of-death, heart disease remained the primary cause, followed by malignant neoplasms. The number of patients with SS-ILD and SS combined with tumors in the past 20 years and the standardized mortality rate after 5 years was higher than that in the first 5 years. Although our results showed that the overall mortality rate of SS was low, an upward trend suggested that clinicians should actively regulate and rationally treat the disease and closely monitor patients with risk factors to improve their prognosis and further reduce the mortality rate.
